# A bacterial enzyme may correct 2-HG accumulation in human cancers

**DOI:** 10.3389/fonc.2023.1235191

**Published:** 2023-07-20

**Authors:** William J. Yin

**Affiliations:** ^1^ Oconee County High School, Watkinsville, GA, United States; ^2^ Bio-Imaging Research Center, The University of Georgia, Athens, GA, United States

**Keywords:** AML, glioma, IDH, 2-hydroxyglutarate, NADPH, 2-HG synthase, nanoparticles, enzyme therapy

## Abstract

A significant proportion of lower-grade glioma as well as many other types of human cancers are associated with neomorphic mutations in *IDH1/2* genes (*mIDH1/2*). These mutations lead to an aberrant accumulation of 2-hydroxyglutarate (2-HG). Interestingly, even cancers without *mIDH1/2* can exhibit increased levels of 2-HG due to factors like hypoxia and extracellular acidity. Mounting evidence demonstrates that 2-HG competitively inhibits α-ketoglutarate dependent enzymes, such as JmjC-domain-containing histone demethylases (JHDMs), ten-eleven translocation enzymes (TETs), and various dioxygenases (e.g., RNA m6A demethylases and prolyl hydroxylases). Consequently, the hypermethylation of DNA, RNA, and histones, and the abnormal activities of hypoxia-inducible factors (HIFs) have profound impacts on the establishment of cancer metabolism and microenvironment, which promote tumor progression. This connection between the oncometabolite 2-HG and glioma holds crucial implications for treatments targeting this disease. Here, I hypothesize that an ectopic introduction of a bacterial 2-hydroxyglutarate synthase (2-HG synthase) enzyme into cancer cells with 2-HG accumulation could serve as a promising enzyme therapy for glioma and other types of cancers. While absent in human metabolism, 2-HG synthase in bacterial species catalyzes the conversion of 2-HG into propionyl-CoA and glyoxylate, two metabolites that potentially possess anti-tumor effects. For a broad spectrum of human cancers with 2-HG accumulation, 2-HG synthase-based enzyme therapy holds the potential to not only correct 2-HG induced cancer metabolism but also transform an oncometabolite into metabolic challenges within cancer cells.

## Introduction

*IDH1* and *IDH2* genes encode TCA-associated isocitrate dehydrogenases, two NADP^+^- dependent enzymes that normally oxidative-decarboxylate isocitrate into alpha-ketoglutarate (α-KG; also called 2-oxoglutarate). Compared to mitochondrial NAD^+^-dependent isocitrate dehydrogenase (encoded by *IDH3* gene), IDH1/2 generate a large amount of NADPH, which is crucial for the REDOX homeostasis in the cytosol and mitochondria.

**Figure 1:**
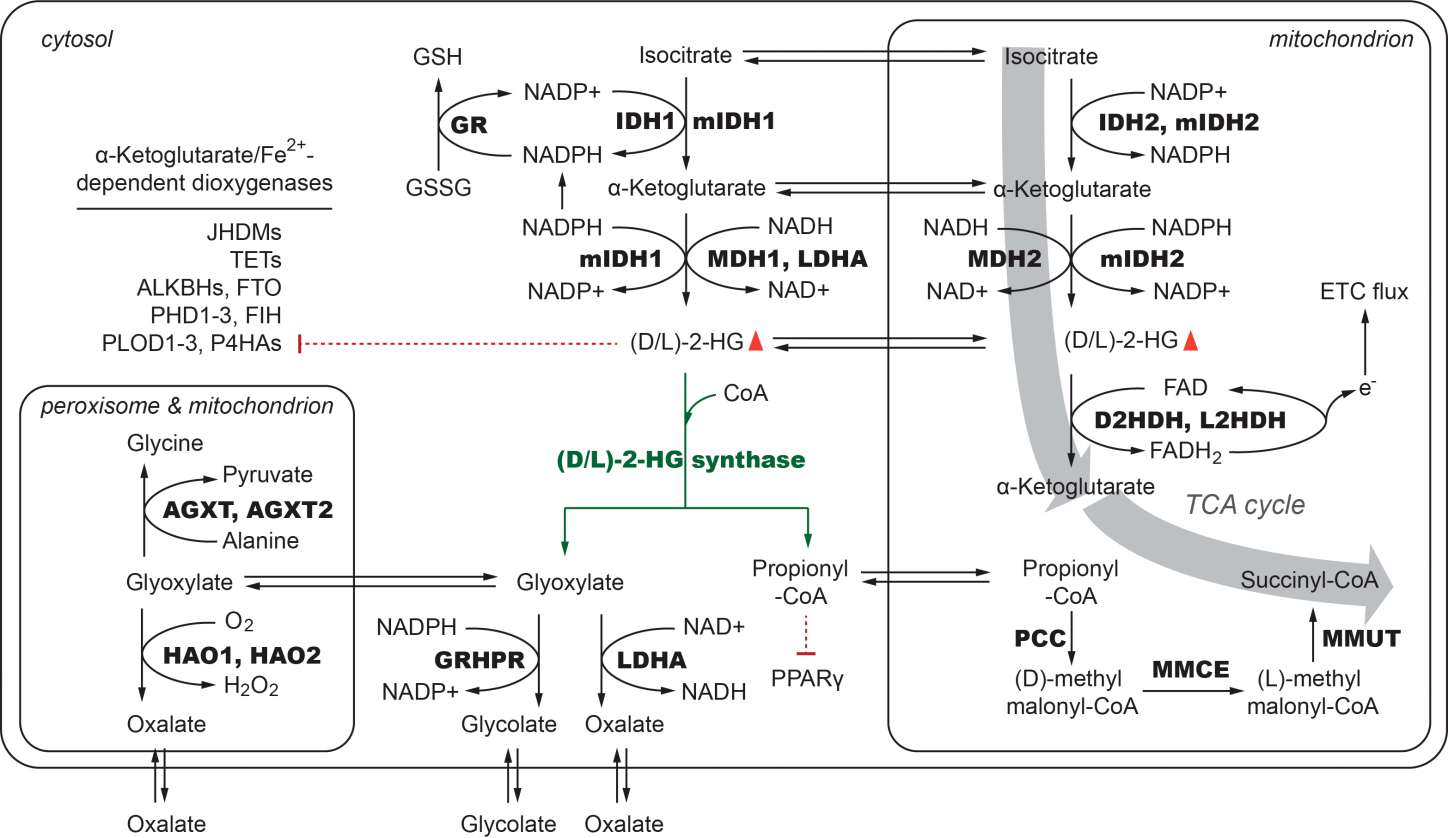
The synthesis and metabolism of 2-HG and its effects mIDH1/2: mutant IDH1 and/or mutant IDH2; IDH1/2, Isocitrate dehydrogenase 1/2; MDH1/2, Malate dehydrogenase 1/2; LDHA, Lactate dehydrogenase A; D2HDH, L2HDH, D-2, Hydroxyglutarate Dehydrogenase, L-2-Hydroxyglutarate Dehydrogenase; GR, Glutathione reductase; PCC, Propionyl-CoA Carboxylase; MMCE, Methylmalonyl-CoA Epimerase; MMUT, Methylmalonyl-CoA Mutase; GRHPR, Glyoxylate Reductase/Hydroxypyruvate Reductase; AGXT1/2, Alanine-glyoxylate Aminotransferase 1/2; HAO1/2, Hydroxy Acid Oxidase 1/2.

About 70-80% of lower-grade glioma and most secondary glioblastoma cases are associated with mutations in the *IDH1* and *IDH2* genes (1). In addition, *IDH1/2* mutations are also found in ~20% of cases of acute myeloid leukemia (AML), ~50% of cases of chondrosarcoma, and ~20% of cases of angio-immunoblastic T-cell lymphoma (2, 3). *IDH1/2* mutations in cancer cases are mostly missense variants, causing a single amino acid substitution of arginine residues at codon 132 in the *IDH1* gene or codons 140/172 in the *IDH2* gene. Inevitably, the above characteristic mutations lead to a neomorphic gain of function of mutated IDH1/2 enzymes (mIDH1/2), which, in addition to the above-mentioned normal catalytic activity, also further catalyze the reduction of α-KG into (D)-2-hydroxyglutarate [(D)-2-HG] (4, 5). As a result, isocitrate is decarboxylated by mIDH1/2 into (D)-2-HG without net production of NADPH (**Figure 1**).

The presence of mIDH1/2 profoundly impacts the metabolism and microenvironments of glioma and AML cancer cells, which can be considered in aspects of both (D)-2-HG accumulation and reduced production of NADPH.

First, the most noticeable metabolic alteration as a result of mIDH1/2 is the aberrant accumulation of (D)-2-HG, which often reaches very high concentrations (5-35 mM) in glioma cells (6). (D)-2-HG structurally resembles α-KG. Thus, (D)-2-HG in its high concentrations acts as a competitive inhibitor for enzymes that utilize α-KG as a substrate. These include JmjC-domain-containing histone demethylase (JHDMs), ten-eleven translocation (TET) enzymes, RNA m6A demethylases, peptidyl prolyl, asparaginyl, and lylyl hydroxylases along with many others. The inhibition of TETs, RNA m6A demethylases, and JHDMs causes hypermethylation of DNA, RNA, and histones and the subsequent global alteration of gene expression. It is noteworthy that α-KG-dependent enzymes have different sensitivities to 2-HG accumulation (for KDM4A/JMJD2A, IC_50 = _24 μM; for KDM4C/JMJD2C, IC_50 = _79 μM; for KDM2A, IC_50 = _106 μM; for KDM5B, *K*_i_ = 10.9 mM; for ALKBH2, IC_50 = _424 μM; for FIH, IC_50 = _1.5 mM; for PHD2, IC_50 = _7.3 mM) (7, 8). As such, the epigenetic alterations in response to mIDH1/2 are quantitatively determined by the levels of 2-HG accumulation and may vary in different cancer types, different cancer stages, and even different subpopulations in cancers.

The impacts of 2-HG on peptidyl prolyl hydroxylases such as PHD2 are complicated and may depend on the specific cellular context, the concentration of 2-HG, as well as specific chiral forms of 2-HG. Generally, (D)-2-HG competitively inhibits PHDs *in vitro* and in many types of cancers with mIDH1/2. However, it was also reported that this oncometabolite enhances PHD2 activity in glioma models and patient samples (9). The non-enzymatic oxidation of 2-HG to α-KG may contribute to such an activation effect (10). PHD activities are negatively associated with the stability of hypoxia-inducible factor (HIF) HIF-1α and HIF-2α, which form transcription complexes with HIF-1β/ARNT and other transcription factors. By this means, the aberrant accumulation of (D)-2-HG may alter the expression levels of hundreds of HIF target genes. An example is the repression of LDHA expression in gliomas carrying *IDH1/2* mutations (11, 12).

(D)-2-HG accumulation also affects the tumor microenvironment. For instance, collagen is a major component in the extracellular matrix and basement membrane. The maturation of collagen requires the hydroxylation modifications of multiple proline and lysine residues, which facilitate the forming of collagen triple helix and the later crosslinking into fibrous collagen. (D)-2-HG competitively inhibits α-KG-dependent procollagen-lysine 2-oxoglutarate 5-dioxygenases PLOD1 and PLOD3 as well as the alpha catalytic subunits of procollagen-proline, 2-oxoglutarate 4-dioxygenases P4HAs (13). These inhibitory effects cause perturbed collagen maturation and instability of the basement membrane, which favors glioma growth and metastasis.

Immune cells are the main players in the tumor microenvironment. Numerous studies demonstrated that tumor cells acquire immune privilege by overexpressing immune checkpoint molecules and suppressing functions of adjacent immune cells (14). Some evidence showed that (D)-2-HG potentiates immunosuppression by decreasing T cell activity. For example, (D)-2-HG, when taken up by activated CD4^+^ cytotoxic T cells through sodium-dependent dicarboxylate transporter SLC13A3, interferes with the transcriptional activities and polyamine synthesis, resulting in inhibition of T cell activity (15). In addition to deactivating local T cells, (D)-2-HG also diminishes the chemotaxis of immune cells to the tumor sites by inhibiting the secretion of C-X-C motif chemokine ligand 10 (CXCL10) by dendritic cells (16). Thus, the immunosuppressive effects of (D)-2-HG also contribute to tumor progression.

As mentioned above, mIDH1/2-induced (D)-2-HG accumulation generally promotes cancer progression and metastasis. This forms the foundation for developing therapeutic agents that specifically target (D)-2-HG accumulation. For example, the recently developed small molecule inhibitors for mIDH1, mIDH2, or both mIDH1/2 have been shown to be capable of efficiently “normalizing” the enzymatic activities of mIDH1/2 (namely inhibiting the activity of reduction of α-KG into (D)-2-HG yet retaining the activity of oxidation of isocitrate into α-KG). This leads to the almost complete abolishment of (D)-2-HG accumulation *in vitro* and *in vivo* (17). FDA recently approved the use of Olutasidenib (a mIDH1 inhibitor) in cases of relapsed or refractory AML, which achieved a ~46% overall response rate in a Phase II clinical trial (18). More clinical trials are ongoing for monotherapies or combination therapies of mIDH1/2 inhibitors LY3410738 and IDH305 for gliomas and other solid tumors with IDH1/2 mutations (ClinicalTrials.gov). A recently finished clinical trial for Olutasidenib monotherapy indicates that the overall response rate is ~8% in a cohort of patients with advanced gliomas and GBM (19).

While correcting (D)-2-HG accumulation has promising therapeutic effects towards malignancies with mIDH1/2, the other aspect of mIDH1/2-elicited metabolic effects, namely the decreased NADPH production, should not be neglected. As mentioned above, mIDH1/2 catalyzes the reduction of α-KG into (D)-2-HG with an electron supply from NADPH leading to no net production of NADPH from the decarboxylation of isocitrate. This would pose a unique challenge for glioma cells with mIDH1/2. It is well known that most cancer cells including glioma are associated with elevated oxidative stress (20). Cancer cells counteract oxidative stress and maintain proliferation by mobilizing a panel of NADPH-dependent enzymatic antioxidants including glutathione reductase (GR) and thioredoxins (TRXs). GR ensures the recycling of the oxidized glutathione (GSSG) back to its reduced form (GSH), which is the essential cofactor for ROS scavenging enzymes (e.g., glutathione peroxidases) to neutralize ROS. On the other hand, TRXs and related peroxiredoxins (PRXs) utilize the reducing power from NADPH to reduce oxidized thiol groups on peptidyl cysteines, which prevents protein dysfunction due to oxidation. Notably, it has been reported that as much as 65% of NADPH produced in the brain originates from joint activities of wildtype IDH1 and IDH2, highlighting the pivotal role of wildtype IDH1/2 activity in the maintenance of REDOX homeostasis in the brain (21). Indeed, multiple lines of studies have demonstrated that mIDH1/2 elicits altered NADPH/NADP^+^ balance in glioma cells, which mount antioxidative defense mechanisms to counteract increased oxidative stress (22–24).

Given the above, it is conceivable that wildtype IDH1/2 activity might be essential for the oxidative stress defense system and the survival/proliferation of cancer cells. In this regard, the presence of mIDH1/2 may also represent a weakness in these tumor cells (in addition to the contributory effects on tumor progression). Several lines of evidence corroborate this notion. First, *IDH1/2* mutations are always monoallelic (25), suggesting the wildtype activities of IDH1/2 are essential to the survival of cancer cells. Second, the wildtype IDH1/2 enzymes have been shown to be overexpressed from the intact alleles in glioblastoma cells carrying *mIDH1/2* (26), implicating a compensatory mechanism in response to the selective pressure of oxidative stress. Also, glioblastoma patients diagnosed with *IDH1/2* mutations usually have a better prognosis and longer mean survival times than patients in *IDH1/2* wildtype cases in the same clinical grades (27). The increased susceptibility to oxidative stress due to mIDH1/2 activities may underlie this clinical trend.

## Expected effects of bacterial 2-HG synthase in cancer cells with mIDH1/2

In human metabolism, (D)-2-HG and its enantiomer (L)-2-HG can be oxidized back into α-KG by mitochondria-located (D/L)-2-HG dehydrogenases (gene symbols: *D2HGDH* and *L2HGDH*). Bi-allelic mutations in D2HGDH and L2HGDH lead to (D/L)-2-hydroxyglutaric aciduria, a group of rare metabolic disorders characterized by the accumulation of (D/L)-2-HG as well as developmental delay, hypotonia, and peripheral neuropathy. The *K*m value of D2HGDH for (D)-2-HG is 0.12 mM, implicating that this enzyme is involved in the metabolism of (D)-2-HG in mIDH1/2-carrying cancer cells. A recent study indicated that D2HGDH is a high-affinity/low-capacity enzyme and its maximum (D)-2-HG degradation rate is likely reached in cancer cells, which still cannot match the 2-HG generation rate of mIDH1/2 (28). As D2HGDH and L2HGDH utilize FAD to relay electrons from (D/L)-2-HG to ETC, the above-observed low capacity may reflect a limitation of ETC flux in these cancer cells, which underlies the aberrant accumulation of (D)-2-HG (**Figure 1**).

As reverting (D)-2-HG accumulation benefits patients with mIDH1/2, **the central concept in this hypothesis is introducing a “bridging” enzyme that converts (D)-2-HG into common metabolizable molecules in cancer cells and hence not only preventing (D)-2-HG accumulation but also reducing the availability of NADPH (i.e., taking advantage of the aberrant activities of mIDH1/2) for better control of tumor progression**.

To find the “bridging” enzyme, Expasy enzyme database was searched for enzymes that utilize 2-HG as a substrate, which returned nine results. The following criteria were applied in narrowing down candidates from the results:

(1) the estimated equilibrium constant of the reaction, which determines the concentration of 2-HG with the presence of the bridging enzyme (prefer a reaction with products in low physiological concentrations to ensure 2-HG concentration can be reduced to a therapeutic meaningful level).
(2) the nature of the new products (ensure the catabolic products of 2-HG can be further metabolized and preferably without negative effects on normal tissues).
(3) the impact on REDOX homeostasis (ensure the catabolic reaction for 2-HG does not generate NADPH).
(4) if available, the *K*m, *K*cat, and specific constant values of the enzyme (prefer an enzyme with high capacity and high affinity to 2-HG).
(5) the selectivity of the enzyme to specific 2-HG enantiomers (prefer an enzyme catabolizing both (D) and (L) forms of 2-HG).

In the above search, a bacterial enzyme 2-HG synthase (EC 2.3.3.11) was found that may serve as a 2-HG catabolizing enzyme under conditions of 2-HG accumulation.

In E. Coli., 2-HG synthase catalyzes the following reversible reaction:


$$\left(L/D\right)-2-hydroxyglutarate\;+\;CoA\;+\;H^{+}\;⇌\;\;propionyl-CoA\;+\;glyoxylate\;+\;H_{2}O\;\;$$

Propionyl-CoA and glyoxylate are two human metabolites that can both be readily metabolized via multiple metabolic pathways. In humans, propionyl-CoA is mainly derived from commensal bacteria fermentation, catabolism of amino acids (isoleucine, methionine, threonine, and valine), and β-oxidation of odd-chain fatty acids (not the major components of lipids in common human diets). Its plasma concentration in healthy humans is very low (<10 μM) (29). In human metabolism, glyoxylate is a toxic intermediate metabolite from the 4-hydroxyproline degradation pathway. Its plasma concentration in healthy humans is about 25 μM (30). Given the stark contrast of high concentrations of (D)-2-HG and CoA with low concentrations of propionyl-CoA and glyoxylate in cancer cells with mIDH1/2, it is expected that the forward reaction (as shown above) would be highly thermodynamically favorable and thus the ectopic presence of 2-HG synthase would convert mIDH1/2-derived (D)-2-HG into propionyl-CoA and glyoxylate in tumor cells.

Both propionyl-CoA and glyoxylate can be further metabolized in glioma cells. In mitochondria, propionyl-CoA can be sequentially converted into (D)-methyl malonyl-CoA, (L)-methyl malonyl-CoA, and succinyl-CoA by propionyl-CoA carboxylase (PCC), methylmalonyl-CoA epimerase (MMCE), and (L)-methyl malonyl-CoA mutase (MMUT). By this means, the overall conversion of isocitrate first into (D)-2-HG and later into succinyl-CoA *de facto* bypasses the TCA cycle step catalyzed by alpha-ketoglutarate dehydrogenase complex. An essential difference between the original steps in the TCA cycle and the bypass lies in that the combined activities of mIDH1/2 and bacterial 2-HG synthase would not generate NADPH and hence would not contribute to the anti-oxidative defense system in tumor cells. Thus, compared to the effects of mIDH1/2 inhibitors, the potential therapeutic effects of 2-HG synthase may include not only the abolishment of (D)-2-HG accumulation but also a decrease in the production of NADPH, which warrants further experimental investigation.

Besides the above therapeutic effects, the generation of propionyl-CoA may have other anti-tumor effects. A recent study reported *in vitro* effects of propionate on activating apoptosis in U87 GBM cells (31). In hepatocellular carcinoma cells, propionyl-CoA represses tumor cell proliferation by inhibiting citrate synthase, leading to reduced TCA flux, mitochondrial respiration, and ATP production (32). In HCT116 colon cancer cells and several other cancer cell lines, propionate induces the cell surface expression of immune stimulatory ligands MICA/B and results in NKG2D-mediated cancer cell killing (33).

Besides propionyl-CoA, 2-HG synthase also generates glyoxylate from 2-HG in a 1:1 stoichiometric ratio. Glyoxylate is a toxic 2-carbon aldehyde, which is very reactive towards amine groups and induces the non-enzymatic formation of advanced glycation end products (AGEs) on proteins. AGE modifications usually result in dysfunctional proteins, which must be removed through proteasome/lysosome-mediated protein degradation. This would expectedly exaggerate the proteostasis burden in tumor/cancer cells. In support of this view, glyoxylate has been reported to have anti-tumor effects in HCT116 colon cancer cells (34). Notably, as (D)-2-HG accumulation is caused by mIDH1/2, 2-HG synthase-derived glyoxylate may have specific toxicity to tumor/cancer cells, but not in adjacent normal cells.

**Figure 2:**
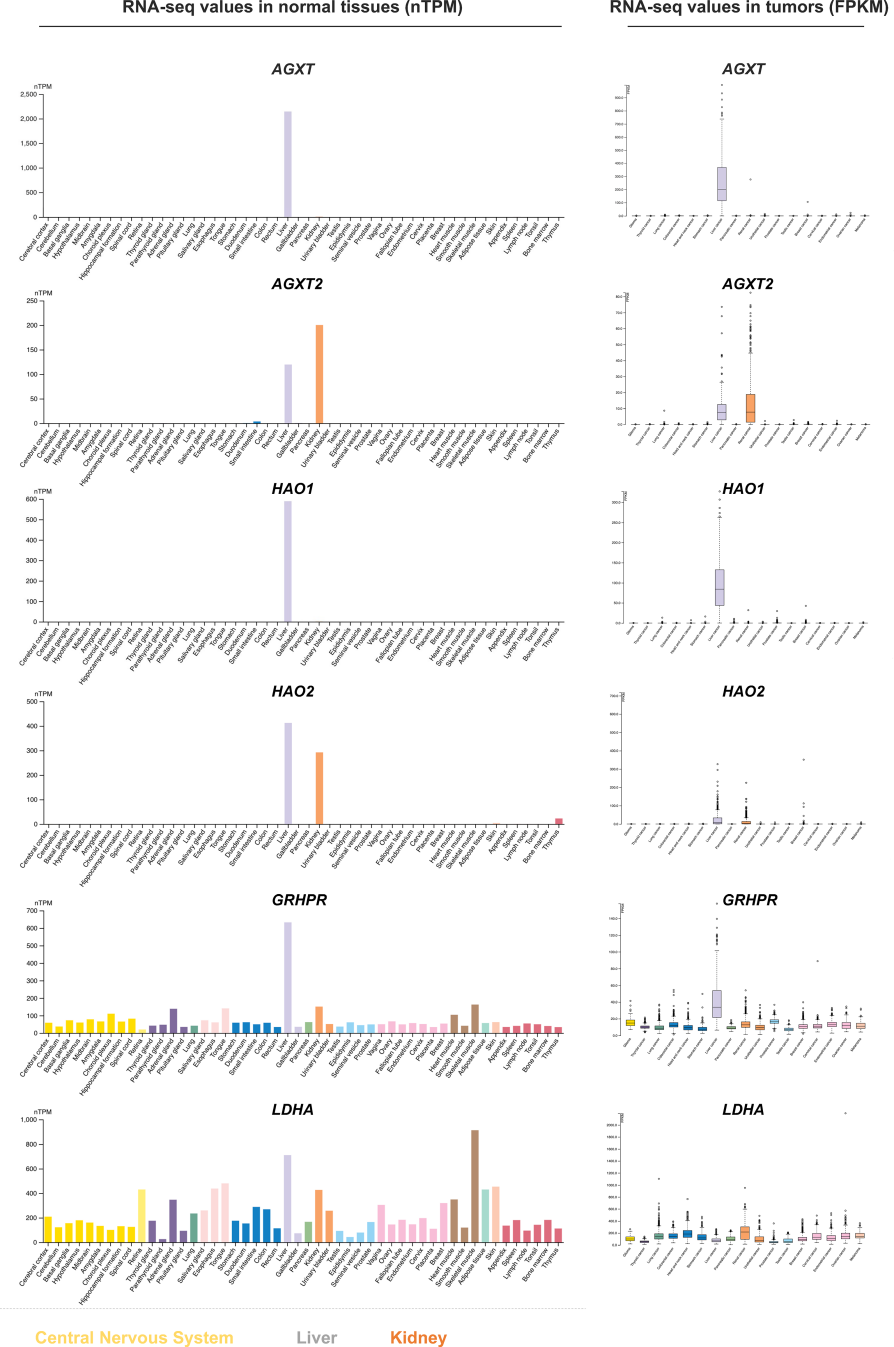
The expression levels of glyoxalate-detoxifying enzymes in normal and tumor tissues. The expression levels were collected from the Human Protein Atlas database (35). Expression values in normal tissues are in nTPM units. Expression values in cancer tissues are in FPKM units. AGXT1/2, Alanine-glyoxylate Aminotransferase 1/2; HAO1/2, Hydroxy Acid Oxidase 1/2; GRHPR, Glyoxylate Reductase/Hydroxypyruvate Reductase; LDHA, Lactate dehydrogenase A.

Given the toxicity of glyoxylate, human cells have detoxifying enzymes that convert glyoxylate into other less toxic and non-toxic metabolites. In hepatocytes, glyoxylate can be effectively metabolized into glycine by aminotransferases, AGXT and AGXT2, present in peroxisome and mitochondria, respectively. Peroxisomes and mitochondria in liver cells also contain hydroxyacid oxidases HAO1 and HAO2, enzymes that oxidize glyoxylate into oxalate. Interestingly, RNA-seq results indicate that the above glyoxylate detoxifying enzymes do not express (or express in extremely low levels) in either glioblastoma/glioma cells or various normal cell types in the brain (**Figure 2**), suggesting that glioma cells cannot detoxify glyoxylate through above metabolic pathways. Besides AGXT/HAO-mediated glyoxylate detoxification, two cytoplasmic enzymes GRHPR and LDHA can catalyze REDOX reactions to convert glyoxylate into glycolate or oxalate, respectively. Both enzymes are ubiquitously present in all normal and tumor tissues and are efficient in glyoxylate metabolism (GRHPR *K*m = 0.24 mM, *K*cat/*K*m = 110/s*mM, LDHA *K*m = 0.18 mM, *K*cat/*K*m = 100/s*mM (36). However, one can speculate that neither of these conversions would benefit solid tumors like gliomas. First, GRHPR reduces glyoxylate into glycolate at the expense of cytoplasmic NADPH, whereby NADPH could be further exhausted in glioma cells. Second, although LDHA can reportedly oxidize glyoxylate into oxalate *in vitro*, such activity of LDHA has not been confirmed in glyoxylate metabolism *in vivo* such as under the condition of hyperoxaluria (37). In addition, if LDHA did oxidize glyoxylate in glioma cells, the generation of cytoplasmic NADH from glyoxylate oxidation would conceivably impede glycolysis, which is a critical energy-generating pathway for glioma cells (38).

## 2-HG synthase-based enzyme therapy may have broad applications in cancer treatment

*IDH1/2* mutations are not found in the vast majority of primary GBM cases, which limits the use of mIDH1/2 inhibitors in this most aggressive form of brain tumor. However, it is notable that the accumulation of oncometabolites (D)-2-HG and (L)-2-HG is much more prevalent than *IDH1/2* mutations in the cancer spectra. Promiscuous substrate usage by LDHA and other REDOX enzymes results in the reduction of α-KG into (L)-2-HG under hypoxia, a condition frequently observed in solid tumors (39). Indeed, it has been reported that elevated (D/L)-2-HG levels can be detected in many types of solid cancers (e.g., breast cancer, colorectal cancer, kidney cancer) independent of mIDH1/2 (40). In addition, elevated (D)-2-HG level is also associated with non-cancer disease conditions, such as cardiomyopathy (41). The concentrations of (D/L)-2-HG in these conditions were observed up to the millimolar range, comparable with the concentrations of (D)-2-HG in mIDH1/2-carrying cancer cells (42). Thus, it is very likely that the elevated (D/L)-2-HG has previously described detrimental effects under the above disease conditions, which would benefit from a reduction in 2-HG concentration.

Intriguingly, mounting evidence shows that many conditions in cancer cells other than *IDH1/2* mutations may lead to an increase in (D/L)-2-HG concentrations. These include, but are not limited to, the reduction of α-KG by MDH1, MDH2, and LDHA, the decreased back-conversion of (D/L)-2-HG into α-KG due to diminished D2HGDH/L2HGDH activities as well as hypoxia and intracellular acidification (40). Conceivably, the above conditions would not be responsive to mIDH1/2 inhibitors yet still benefit from 2-HG synthase treatment that targets 2-HG accumulation directly rather than specific enzyme activities. This implies that 2-HG synthase-based enzyme therapy may have a broad scope of applications for cancers with elevated 2-HG concentrations by converting the excess (D/L)-2-HG into metabolites with anti-tumor effects.

## Targeted therapeutic delivery via functionalized nanoparticles

Although 2-HG may accumulate in very high concentrations in various disease conditions, 2-HG in relatively low levels has also been detected in normal cells, which can be generated and degraded by a panel of human enzymes under physiological conditions (43). In fact, recent studies indicate that 2-HG, as a reduced form of α-KG, may be integral to human metabolism and play elaborate roles in concert with various α-KG-dependent dioxygenases in metabolic regulation. For instance, hypoxia induces the production of (L)-2-HG, which is sufficient to enhance global H3K9me3 modifications in glioblastoma cells due to the inhibitory effect of 2-HG on histone demethylase KDM4C (39). In addition, CD8^+^ T lymphocytes have been found to generate (L)-2-HG in response to TCR activation and hypoxia, which promotes cell proliferation and survival (44).

As ectopic 2-HG synthase may disturb physiological functions of 2-HG in normal cells, the delivery of therapeutic 2-HG synthase in its DNA, RNA, or protein forms entails possible side effects and thus tumor cell-specific targeted delivery is preferred. Recent advances in nanoparticles functionalized with cell-type specific targeting properties and blood-brain barrier (BBB) permeability shed light on the future development of 2-HG synthase-based enzyme therapies (45).

Organic nanoparticles that are fabricated from natural or synthetic polymers and lipids are by far the most used nanocarriers for therapeutics in DNA, RNA, and protein forms in experimental and clinical settings. Organic nanoparticles are biodegradable and biocompatible and can be functionalized with antibodies, peptides, and metabolites on the surface to facilitate targeted tumor-specific delivery and BBB permeability (46). For example, poly(lactic acid) (PLA) nanoparticles modified with surface CREKA peptides (cysteine–arginine–glutamic acid–lysine–alanine; with high affinity to fibrin) or Ft peptides (synthetic FHK, cysteine, and tLyp-1 sequence; with high affinity to tenascin C and neuropilin 1) have been experimentally proved to effectively target glioblastoma/glioma models *in vivo* (47, 48). In another study, PLA and hyperbranched polyglycerol hybrid polymer nanoparticles were shown to successfully deliver apoptosis-inducing microRNA-21 to the intracranial glioma site (49). Besides PLA, poly (lactic-co-glycolic acid) (PLGA) is another extensively studied nanoparticle polymer for therapeutic delivery. In one study, PLGA nanocarriers loaded with paclitaxel were further functionalized with superparamagnetic iron oxide nanoparticles, which achieved magnetic-guided glioblastoma targeting in the brain (50). Lipid-based nanoparticles (liposomes) are a classic type of nanocarrier for gene/enzyme therapy. The surface of liposomes can be functionalized with targeting ligands (e.g., transferrin or anti-transferrin receptor single-chain antibodies) to facilitate tumor-specific delivery, or with cations, polyethylene glycol, or antibodies to aid BBB crossing. Multiple studies demonstrated that low-intensity focused ultrasound can induce transient opening of BBB, which in combination with intravenous injection of tumor-targeting liposomes can accomplish prolonged glioma suppression (51, 52).

It is worthy of mention that recently developed DNA/RNA-based nanoparticles (aka. flowers) may be the next-generation therapeutic delivery tools for glioblastoma (53). Based on Watson-Crick base-pairing, single-stranded DNA/RNA can be designed to self-assemble into three-dimensional structures in different shapes and sizes. A unique advantage of DNA/RNA nanomaterials is that the rich information embedded in DNA/RNA sequences can be utilized to include aptamer sequences for cell-type specific targeting and anti-sense sequences for biosensing (54). Thus, the release of the cargo in DNA/RNA nanoparticles can be elaborately controlled by specific cell types and selective cellular states (e.g., the expression of certain genes) (55). DNA and RNA aptamers for glioblastoma have been reported before (56). In the future, the artificial intelligence-aided discovery of riboswitch that is responsive to metabolites such as 2-HG may be incorporated into the design of DNA/RNA nanoparticles that release 2-HG synthase specifically in tumor cells with 2-HG accumulation.

## Conclusion and perspective

Glioblastoma/glioma and many other types of solid tumors/cancers are associated with aberrant 2-HG accumulation due to altered activities of α-KG-related REDOX enzymes. The accumulation of 2-HG as a key oncometabolite drives cancer progression via metabolic reprogramming and tumor microenvironment remodeling. mIDH1/2-specific small molecule inhibitors revert the aberrant enzymatic activity and hold promise to treat mIDH1/2-carrying malignancies. However, an alternative possibility also exists wherein instead of preventing (D/L)-2-HG generation, these tumor-cell specific oncometabolites can be metabolized and converted into other molecules. Here, I propose that the administration of bacterial 2-HG synthase could serve as a therapeutic enzyme for solid tumors with (D/L)-2-HG accumulation. The ectopic introduction of bacterial 2-HG synthase may take advantages of the aberrant (D/L)-2-HG accumulation in tumor cells and transform (D/L)-2-HG into anti-tumor metabolites propionyl-CoA and glyoxylate. The continuous generation of propionyl-CoA and glyoxylate from (D/L)-2-HG may mount unique metabolic challenges on cancer cells, particularly the decreased production of NADPH and the consequential oxidative stress.

In this hypothesis, many above-mentioned effects of bacterial 2-HG synthase are speculative and await experimental validation. Given distinct intracellular environments in *E. Coli* and human cancer cells, the enzymatic activity/efficiency, substrate specificity/affinity, protein stability/turnover rate, and possible moonlighting functions of bacterial 2-HG synthase need to be thoroughly examined and optimized in cancer-relevant experimental settings in order to reach therapeutic effects. In particular, the potential therapeutic efficacy of 2-HG synthase relies on the *K*m of the enzyme to its substrates 2-HG, propionyl-CoA, and glyoxylate as well as its kinetic parameters *K*cat and specificity constant. Further modifications of the enzymatic activity by bioengineering approaches might be needed. Besides, the metabolic impacts and potential toxicity of 2-HG-derived glyoxylate should be closely investigated and longitudinally monitored *in vivo*. In addition, the systemic and local immunity in response to the introduction of a bacterial enzyme and the associated indirect effects should be characterized in model organisms before its application in humans.

Due to their sizes and charges, 2-HG synthase and its encoding DNA/RNA molecules cannot pass through the blood-brain barrier. Intrathecal administration may be a necessary route for the enzyme therapeutics to reach glioma sites. Besides, targeted delivery of 2-HG synthase to 2-HG accumulated cells may be essential due to possible disturbance of physiological functions of 2-HG in normal cells. The recent advances in nanocarriers functionalized with targeting ligands may facilitate the tumor-specific delivery of the enzyme therapeutics as monotherapies or combination therapies. However, these therapeutic approaches develop, it is the hope of the author that this hypothesis may lead to a new angle of future research, which may eventually benefit patients with gliomas and many other types of cancers.

## Data availability statement

The original contributions presented in the study are included in the article. Further inquiries can be directed to the corresponding author.

## Author contributions

WY generated the idea and contributed solely to the manuscript preparation.
